# Quercetin-Rich Guava (*Psidium guajava*) Juice in Combination with Trehalose Reduces Autophagy, Apoptosis and Pyroptosis Formation in the Kidney and Pancreas of Type II Diabetic Rats

**DOI:** 10.3390/molecules21030334

**Published:** 2016-03-10

**Authors:** Chia-Fa Lin, Yen-Ting Kuo, Tsung-Ying Chen, Chiang-Ting Chien

**Affiliations:** 1 Department of Life Science, College of Science, National Taiwan Normal University, Taipei 11677, Taiwan; drlin691168@gmail.com (C.-F.L.); sabrina86327@hotmail.com (Y.-T.K.); 2 Department of Cardiology, Kuang-Tien General Hospital, Taichung 433, Taiwan

**Keywords:** apoptosis, autophagy, quercetin, trehalose, oxidative stress, pyroptosis, Type II diabetes

## Abstract

We explored whether the combination of anti-oxidant and anti-inflammatory guava (*Psidium guajava*) and trehalose treatment protects the kidney and pancreas against Type II diabetes (T2DM)-induced injury in rats. We measured the active component of guava juice by HPLC analysis. T2DM was induced in Wistar rats by intraperitoneal administration of nicotinamide and streptozotocin and combination with high fructose diets for 8 weeks. The rats fed with different dosages of guava juice in combination with or without trehalose for 4 weeks were evaluated the parameters including OGTT, plasma insulin, HbA1c, HOMA-IR (insulin resistance) and HOMA-β (β cell function and insulin secretion). We measured oxidative and inflammatory degrees by immunohistochemistry stain, fluorescent stain, and western blot and serum and kidney reactive oxygen species (ROS) by a chemiluminescence analyzer. High content of quercetin in the guava juice scavenged H_2_O_2_ and HOCl, whereas trehalose selectively reduced H_2_O_2_, not HOCl. T2DM affected the levels in OGTT, plasma insulin, HbA1c, HOMA-IR and HOMA-β, whereas these T2DM-altered parameters, except HbA1c, were significantly improved by guava and trehalose treatment. The levels of T2DM-enhanced renal ROS, 4-hydroxynonenal, caspase-3/apoptosis, LC3-B/autophagy and IL-1β/pyroptosis were significantly decreased by guava juice and trehalose. The combination with trehalose and guava juice protects the pancreas and kidney against T2DM-induced injury.

## 1. Introduction

Type II diabetes (T2DM), a kind of metabolic disorder, has become a major problem in a growing number of patients and is showing increasing prevalence both in developed and developing countries [1,2,3,4]. The impairment in insulin secretion by β-cell dysfunction and insulin action by increased insulin resistance contributes to T2DM-induced hyperglycemia [5]. Hyperglycemia induced diabetic nephropathy is the leading cause of chronic kidney disease in patients being prescribed renal replacement therapy and is associated with increased cardiovascular mortality [6,7]. Diabetic nephropathy is characterized by an abnormal level (30 mg/day or 20 g/min) of albumin (microalbuminuria) in urine subsequently leading to proteinuria and end-stage renal failure [8]. Therefore, it is imperative to further understand the pathophysiologic mechanisms and to develop novel strategies to preserve pancreatic β-cell function and to decrease hyperglycemia-induced diabetic nephropathy.

Increasing reactive oxygen species (ROS) which augment oxidative stress in the tissues are generated by hyperglycemia-induced toxicity [6]. Oxidative stress has been considered to be the major factor in diabetic nephropathy. Both intracellular and extracellular hyperglycemia leads to ROS generation and causes tissue damage. High extracellular glucose levels increase the formation of advanced glycation end products (AGE) and activate AGE receptor-mediated signaling to impair cells and tissues/organs [6,7,8]. Enhanced intracellular glucose level promotes the activity of protein kinase C and NADPH oxidase to elevate ROS generation and some transcription factors expression, such as nuclear factor kappa B [9].

Guava is a kind of tropical fruit, popular in Asia, including Taiwan [10]. Guava has anti-oxidative, anti-inflammantory and anti-diabetic properties [11], due to an abundance of vitamin C, flavonoids and polyphenolic compounds [12]. In a previous study, guava extracts could decrease ROS, interlukin-6, tumor necrosis factor-α, and interlukin-1β levels in type 1 diabetic mice kidney [10]. There are several reports on the relationship between gauva leaf extracts and T2DM [13], but few report any relationship between the edible proportion of guava and T2DM.

Trehalose is a disaccharide composed by two glucose units in an α,α-1,1-glycosidic linkage [14]. Trehalose is a natural disaccharide present in a wide variety of organisms, including plants, bacteria, yeast, and invertebrates [15]. This sugar provides protection in cells against environment stress, such as heat, cold, desiccation, dehydration, and oxidation by preventing protein denaturation [16]. Because of its unique properties, trehalose has been used in Huntington’s disease [17], and Alzheimer’s disease [16]. Trehalose can also greatly improve the survival of mammalian cells during cryopreservation [18]. The goal was to explore the effects and mechanisms of guava juice combined with trehalose on the pathophysiology of kidney and pancreas in rats with T2DM.

## 2. Results

### 2.1. Quercetin Content in Guava Extraction

**Figure 1:**
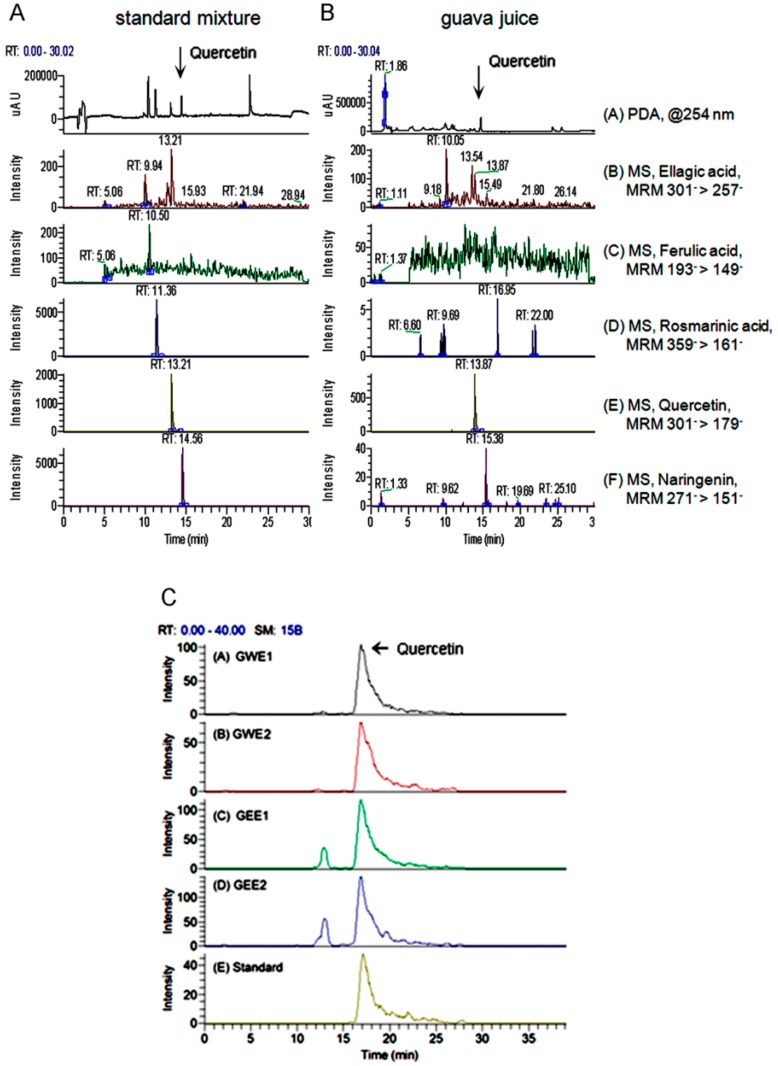
Typical chromatogram of quercetin content in the extract of guava by HPLC analysis. (**A**) The chromatogram of the standard mixture is shown in the left panel (**A**), whereas the chromatogram of the water guava extract is indicated in the right panel (**B**); Five chemicals including ellagic acid, ferulic acid, rosmarinic acid, quercetin and narinegenin are identified in the standard mixture. Only quercetin is found in the guava juice; (**C**) Quercetin content in the extract of guava juice by HPLC analysis.

The guava juice used in this study was prepared from two kinds of guava harvested in Taiwan, Thailand guava (G1) and pearl guava (G2). We tested the content of quercetin, a major anti-oxidative flavonoid in the guava water extract of G1 (GWE1), G2 (GWE2) and guava ethanol extract of G1 (GEE1), G2 (GEE2), respectively. In the LC/MS results, when compared to the standards (Figure 1A), only the quercetin content in guava juice was identified in the Figure 1B. The concentrations of quercetin were 182.3 ng/mL in GWE1, 133.4 ng/mL in GWE2, 223.1 ng/mL in GEE1 and 244.5 ng/mL in GEE2 (Figure 1C). According to guava concentration and volume, we calculated the content of quercetin in our guava juice sample was 0.633 μg/mL. In our study, each group of rats had consumed different doses of quercetin per day. CON (Control) group consumed 0 μg/kg BW, DM (type II DM) group consumed 0 μg/kg BW, B1 rats (T2DM with 4 mL/kg BW of guava juice without trehalose) consumed 2.5 μg/kg BW, T1 (T2DM with 4 mL/kg BW of guava juice with 2 mL/kg BW of trehalose) consumed 2.5 μg/kg BW, T2 rats (T2DM with 8 mL/kg BW of guava juice with 4 mL/kg BW of trehalose) consumed 5.0 μg/kg BW and T5 (T2DM with 20 mL/kg BW of guava juice with 1 mL/kg BW of trehalose) consumed 12.6 μg/kg BW per day.

### 2.2. Guava Juice in Vitro ROS Level

**Figure 2:**
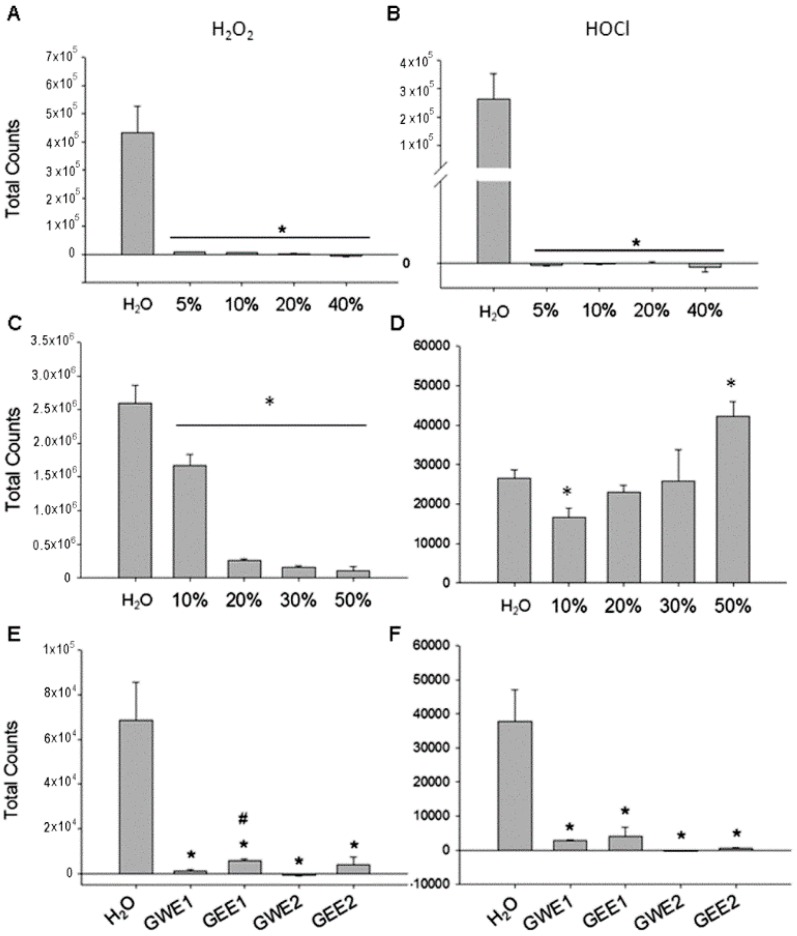
The effect of different concentration of guava juice (**A**,**B**), trehalose (**C**,**D**) and different kinds of guava extraction (**E**,**F**) on scavenging ROS levels of H_2_O_2_ and HOCl *in vitro*. *n* = 3–5 in each group. Water extract of Thailand guava, GWE1; Water extract of pearl guava, GWE2; ethanol extract of Thailand guava, GEE1; ethanol extract of pearl guava, GEE2. * *p* < 0.05 *vs.* H_2_O. # *p* < 0.05 GEE1 *vs.* GWE1.

We used H_2_O_2_ and HOCl as exogenous ROS sources to evaluate the scavenging ROS ability of guava juice. Our results showed that guava juice showed highly and dose-dependently scavenging H_2_O_2_ and HOCl ability from concentration 5% to 40%. (Figures 2A,B).

### 2.3. Trehalose in Vitro ROS Levels

The ability of trehalose to scavenge ROS seemed to be selectively. Trehalose selectively and dose-dependently decreased H_2_O_2_ counts from concentration 10%–50% (Figure 2C). In contrast, trehalose did not affect HOCl counts from concentration 10%–50% (Figure 2D).

### 2.4. Guava Extraction in Vitro ROS Levels

Our results showed that water extract (50 g guava/150 mL H_2_O) and ethanol extract (50 g guava/150 mL ethanol) of two kinds of guava (G1 and G2) displayed high ability to scavenge H_2_O_2_ (Figure 2E) and HOCl ROS (Figure 2F). Using double distilled H_2_O (ddH_2_O) as a reference control, the increased H_2_O_2_ and HOCl counts were significantly (*p* < 0.05) decreased by the four kinds of guava extract. GWE1 and GWE2 were more efficient (*p* < 0.05) than GEE1 and GEE2 in scavenging H_2_O_2_ and HOCl amount according to the luminol-amplified chemiluminescence method. Water extract of guava had a higher ROS scavenging ability than ethanol extract.

### 2.5. Oral Guava Juice Tolerance Test

According to our data, to determine the effects of the three kinds of guava juice on blood glucose levels, we treated normal animals with 4 mL/kg BW guava juice containing 12% trehalose, 8.8% sucrose, or 40% guava juice (without sugar supplement). The three kinds of guava juice treatment did not increase blood glucose dramatically. Additionally, there was no significant difference among them.

### 2.6. Intravenous Glucose and Trehalose Tolerance Test

**Figure 3:**
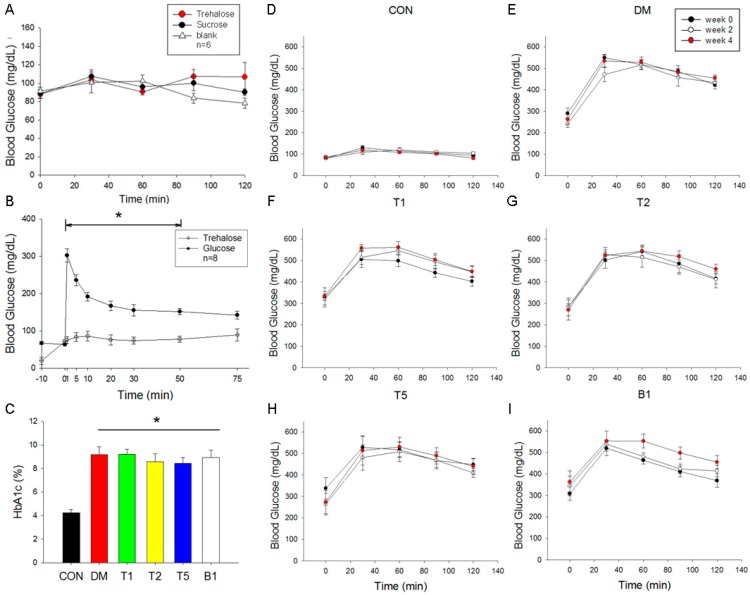
Effect of guava juice and trehalose on glycemic parameters. (**A**) Effect of guava juice and trehalose on oral glucose tolerance test. Normal rats were orally intake 40% guava juice containing 12% trehalose (red), 8% sucrose (black) or ddH_2_O (Blank, white); (**B**) Intravenous glucose and trehalose tolerance test. Normal animals were given 0.5 g/kg glucose (black) or trehalose (white) via an intravenous route. *n* = 8 in each group; (**C**) HbA1c Level. *n* = 3–5 in each group; (**D**–**I**) Oral glucose tolerance test. *n* = 3–5 in each group. Data are expressed as mean ± SEM. * *p* < 0.05 *vs.* CON (control group).

To evaluate the alterations of blood glucose level in response to intravenous (i.v.) glucose or trehalose, we administered 0.5 g/kg body weight glucose or trehalose via an intravenous route. The blood glucose level increased significantly to 302.4 ± 40.4 mg/dL (*n* = 8) at 1 min after i.v. glucose (Figure 3B). However, the blood glucose was not significantly elevated at 1 min after i.v. trehalose. After 1st minute of glucose administration, the elevated blood glucose level started to decrease. However, at 75th min, the blood glucose (143.5 ± 19.5 mg/dL) was still significantly higher (*p* < 0.05) than fasted blood glucose (81.8 ± 3.0 mg/dL) at the time of beginning. On the other hand, normal animals treated trehalose did not show significant changes in blood glucose.

### 2.7. HbA1c Levels

HbA1c level of DM rats was twice that of CON rats. In T2 and T5 rats, HbA1c levels were slightly lowered, though there were no statistically differences (Figure 3C).

### 2.8. Oral Glucose Tolerance Test (OGTT)

In CON rats, the baseline blood glucose levels were around 90–100 mg/dL at week 0, 2 or 4 (Figure 3D). The values of blood glucose in response to OGTT of the CON group were kept around 100–150 mg/dL at week 0, 2 and 4. In DM, T1, T2, T5 and B1 groups, the baseline levels of blood glucose were significantly elevated to 250–360 mg/dL compared to CON group (Figures 3E–I). In response to OGTT, the values of blood glucose were highest at 30 and 60 min and ranged from 500 to 600 mg/dL in these 5 groups with T2DM. The increased levels of blood glucose in response to OGTT were not affected by the supplementary guava juice and trehalose (T1, T2 and T5 groups) or guava juice alone (B1 group).

### 2.9. Blood Glucose Changes

**Figure 4:**
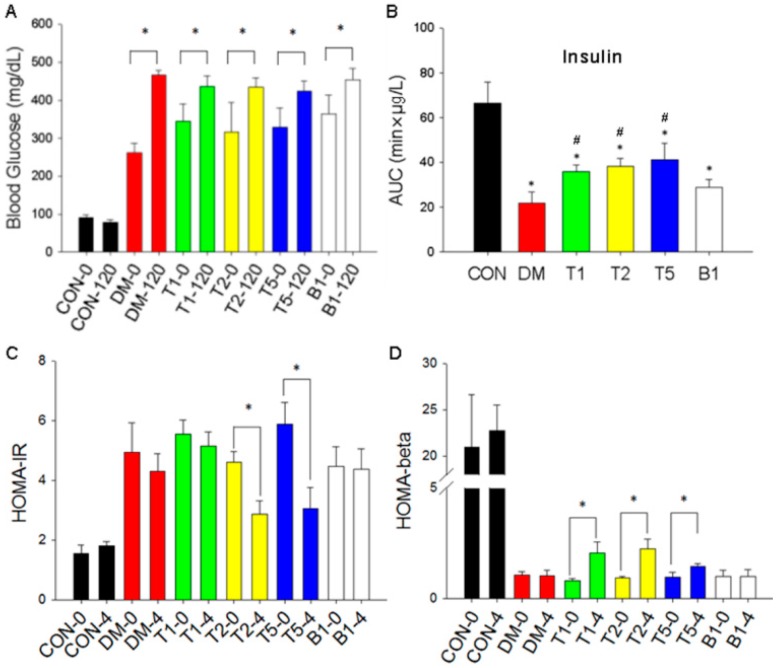
Effect of guava juice and trehalose on OGTT (**A**); insulin levels (**B**); HOMA IR (**C**) and HOMA-β (**D**) in six groups of rats at week 0 and week 4. Data are expressed as mean ± SEM and analyzed by two-way ANOVA. *n* = 3–5 in each group. * *p* < 0.05 *vs.* the value at week 0 of respective group. # *p* < 0.05 *vs.* DM.

We compared FBG (0 min) and blood glucose at the end (120 min) of OGTT test at week 4 (Figure 4A). In CON rats, values of blood glucose at 0 and 120 min were both below 100 mg/dL. There was no difference between 0 min and 120 min. In DM rats, 0 min blood glucose and 120 min was significantly different (*p* < 0.05) because blood glucose couldn’t decline at the end of OGTT test. In T1, T2, T5 and B1 groups, the blood glucose level was significantly higher at 120 min as compared to that at 0 min by two-way ANOVA indicating that treatment of guava juice helped control blood glucose in OGTT.

### 2.10. Insulin Levels

Insulin levels were indicated by the index of AUC (area under curve = min × μg/L) (Figure 4B). AUC of insulin levels were 66.6 ± 2.1 min × μg/L in CON. Due to the impairment of β cells in T2DM, AUC declined to 21.9 ± 1.4 min × μg/L in DM. AUC was elevated in T1 (36.0 ± 0.5 min × μg/L), T2 (38.2 ± 1.1 min × μg/L), and T5 (41.5 ± 4.5 min × μg/L). AUC of B1 was 28.9 ± 0.8 min × μg/L. Guava juice combined with trehalose significantly preserved the insulin secretion function in T2DM rats

### 2.11. HOMA-IR

HOMA-IR values of CON rats were about 2 at week 0 and week 4 (Figure 4C). In DM rats, this value was elevated (up to 4.9 ± 0.2) and remained stable from week 0 to week 4. In T1, T2, T5 and B1 rats, HOMA-IR were all lower at week 4 than week 0. In T2 and T5, these changes were significantly (*p* < 0.05) decreased by using two-way ANOVA. The dose of guava juice and trehalose treated in T2 and T5 rats may not only help preserve the function of insulin secretion, but also lowered the insulin-resistance value. 

### 2.12. HOMA-β

In CON rats, the values of HOMA-β were about 20 at week 0 and week 4 (Figure 4D). All the rats with T2DM had lower HOMA-β values (below 5) compared to CON. The low HOMA-β values in DM rats still remained between week 0 and week 4. However, in T1, T2 and T5 not B1 group, the HOMA-β value was significantly (*p* < 0.05) elevated at week 4 compared to week 0 and DM group by two-way ANOVA. 

### 2.13. HE (Hematoxylin and Eosin) Stain

**Figure 5:**
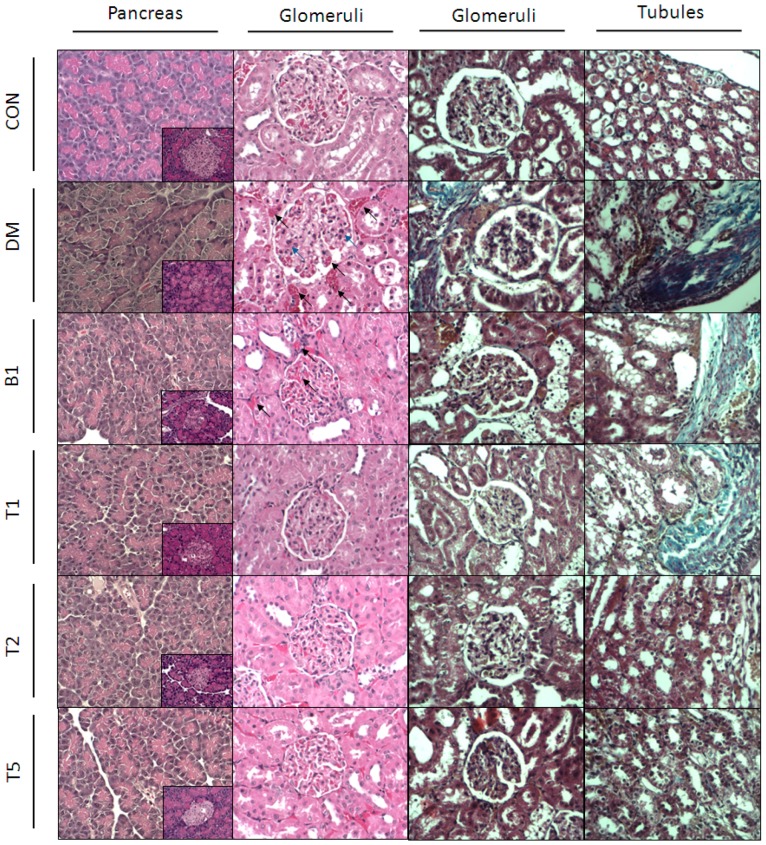
The typical graphs of H & E (left two panels) and Masson’s stains (right two panels) in the pancreas and kidney from six groups of rats.

In pancreatic section, we found that DM rats had smaller islets of Langerhans diameters (Figure 5) implicating islet shrinkage. Besides, pancreas of DM rats showed irregular arrangement. The boundary of cells were blurred or vanished and cells were necrotic. Larger sizes of islet were found in T1, T2, T5, and B1 rats compared to DM rats. Additionally, the arrangement of pancreatic cells in T1, T2, T5, and B1 rats became more regular than DM rats. In renal sections, hemorrhage presented in DM rats. Also, neutrophils occurred in renal section of DM rats. T1, T2, T5 and B1 rats showed less hemorrhage and neutrophils gathering, which meant the inflammatory damage of T2DM on kidney had been rescued. T1, T2 and T5 rats showed less of these characters than B1 rats.

### 2.14. Masson’s Stain

Masson’ stain could stain sclerotic area in a blue color. We found that sclerosis occurred mostly in renal tubules in our T2DM rats rather than glomeruli (Figure 5). T1, T2, T5 and B1 rats showed less blue area in renal sections *vs.* DM. T2 and T5 rats displayed relatively less sclerotic area *vs.* B1.

### 2.15. IHC and Fluorescent Stain in Kidney

**Figure 6:**
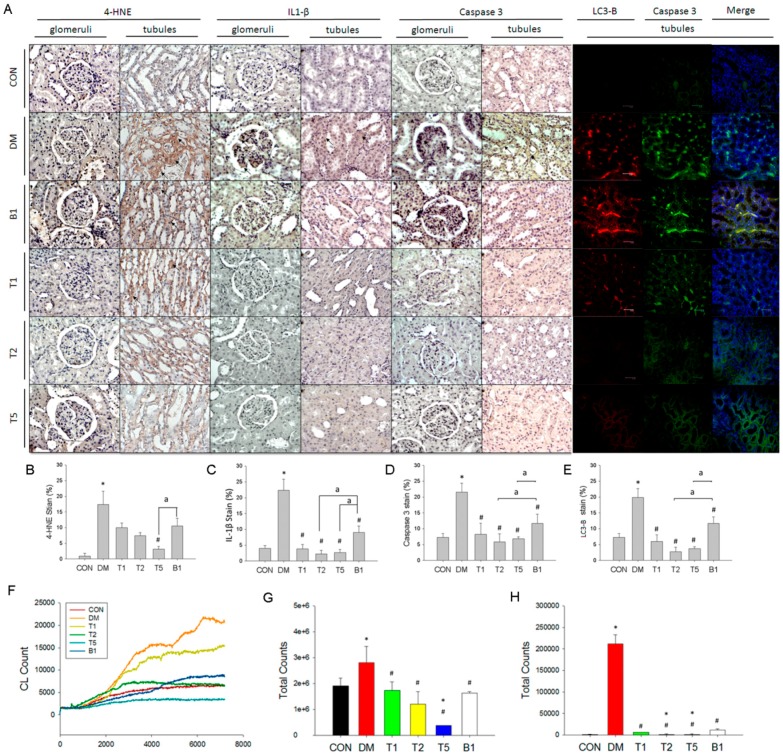
The typical graph of expression (brown area indicated by arrows) of 4-HNE, IL-1β, caspase 3 in renal glomeruli and tubules, and fluorescent LC3-B (red color) and caspase 3 (green color) in renal tubules of six groups (**A**). The statistical data of 4-HNE (**B**); IL-1β (**C**); caspase 3 (**D**) and LC3-B (**E**) are demonstrated in these six groups; (**F**) The original curves show the real-time data of renal ROS during 7200 s of recording in 6 groups of rat kidneys. Statistic data of renal ROS *in vivo* (**G**) and serum ROS levels *in vitro* (**H**) are indicated. Data are expressed as mean ± SEM. *n* = 3–5 each group. * *p* < 0.05 *vs.* CON, # *p* < 0.05 *vs.* DM, ^a^ *p* < 0.05 *vs.* B1.* *p* < 0.05 *vs.* CON. # *p* < 0.05 *vs.* DM. ^a^ *p* < 0.05 *vs.* B1.

Tubular 4-HNE (4-hydroxynonenal, a marker of oxidative stress and lipid peroxidation), IL-1β (pyroptosis marker) and caspase 3 (apoptosis marker) expression were higher in DM rats compared to CON (Figure 6). Rats with guava juice combination with (T1, T2, or T5) or without trehalose (B1) had less tubular 4-HNE, IL-1β and caspase 3 expression. B1 rats had the highest level of 4-HNE, IL-1β and caspase 3 expression compared to T1, T2 and T5.

Green fluorescent represented caspase 3-mediated apoptosis, whereas red fluorescent represented LC3-B-mediated autophagy. In the renal section, high expression of caspase 3 and LC3-B in cytoplasm of DM group compared to CON group (Figure 6). We also found a colocalization of these two proteins in the renal tubules of DM kidney. The level of expressions of caspase 3 and LC3-B proteins were markedly reduced in T1, T2, T5 and B1.

### 2.16. Renal in Vivo ROS Levels

Original (Figure 6F) and statistic data (Figure 6G) showed renal CL count was highest in DM rats. T1, T2, T5 and B1 had lower CL counts *vs.* DM.

### 2.17. Serum in Vitro ROS Level

Serum of DM had higher ROS level significantly higher than that in CON (*p* < 0.05) (Figure 6H). CL counts of T1, T2, T5 and B1 rats were all significantly reduced than those in DM (*p* < 0.05). In addition, T1, T2 and T5 had lower ROS levels than B1. The difference between T1 and B1 was not significant, whereas T2 and T5 had significantly lower serum ROS than B1 (*p* < 0.05).

### 2.18. IHC and Fluorescent Stain in Pancreas

**Figure 7:**
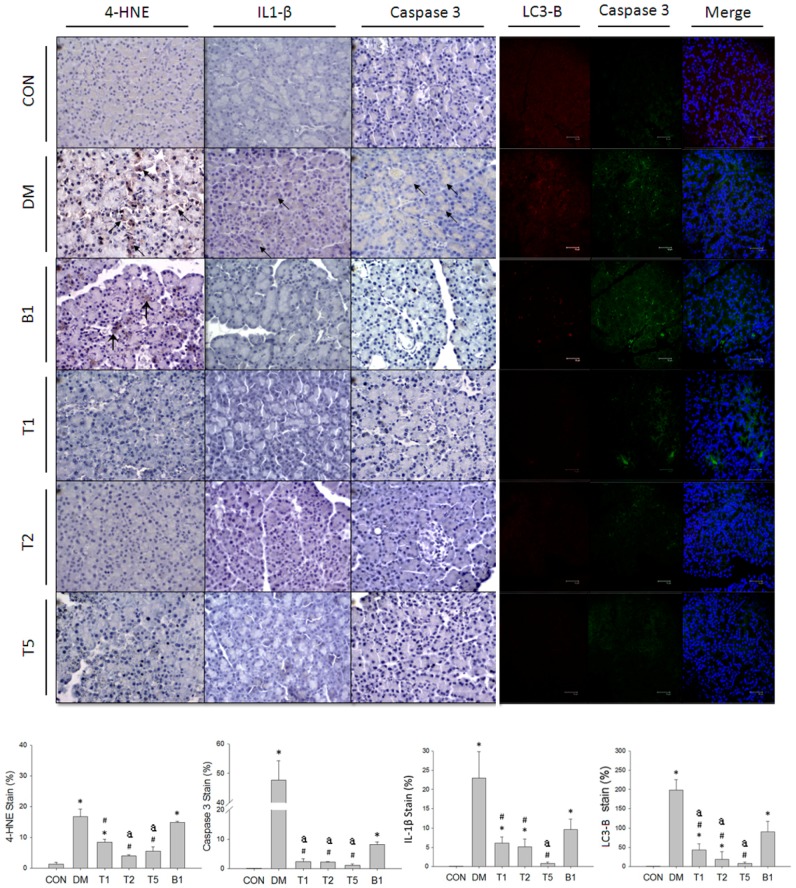
The typical graph of pancreatic expression (brown area indicated by arrows) of 4-HNE (lipid peroxidation and oxidative stress marker), IL-1β (pyroptosis marker), caspase 3 (apoptosis marker) and fluorescent LC3-B (red color, autophagy marker) and caspase 3 (green color) in pancreas of six groups. Data are expressed as mean ± SEM. *n* = 3–5 each group. * *p* < 0.05 *vs.* CON. # *p* < 0.05 *vs.* DM. ^a^ *p* < 0.05 *vs.* B1.

The pancreatic 4-HNE, IL-1β and caspase 3 expressions were significantly higher in DM rats compared to CON rats (Figure 7). T1, T2, T5, or B1 significantly depressed pancreatic 4-HNE, IL-1β and caspase 3 expressions. Furthermore, T1, T2 and T5 were better than B1 rats in reducing pancreatic 4-HNE, IL-1β and caspase 3 expressions.

Higher caspase 3 and LC3-B expressions were also found in pancreatic section of DM rats (Figure 7). Caspase 3 showed higher expression than LC3-B suggesting apoptosis might occur more than autophagy in T2DM. T1, T2 and T5 decreased the expression of caspase 3 and LC3-B in the pancreatic section of T2DM rats. B1 showed higher caspase 3 and LC3-B expression than T1, T2 or T5 implicating the addition of trehalose in guava juice confers further protection against DM-induced pancreatic injury.

### 2.19. Western Blotting 

**Figure 8:**
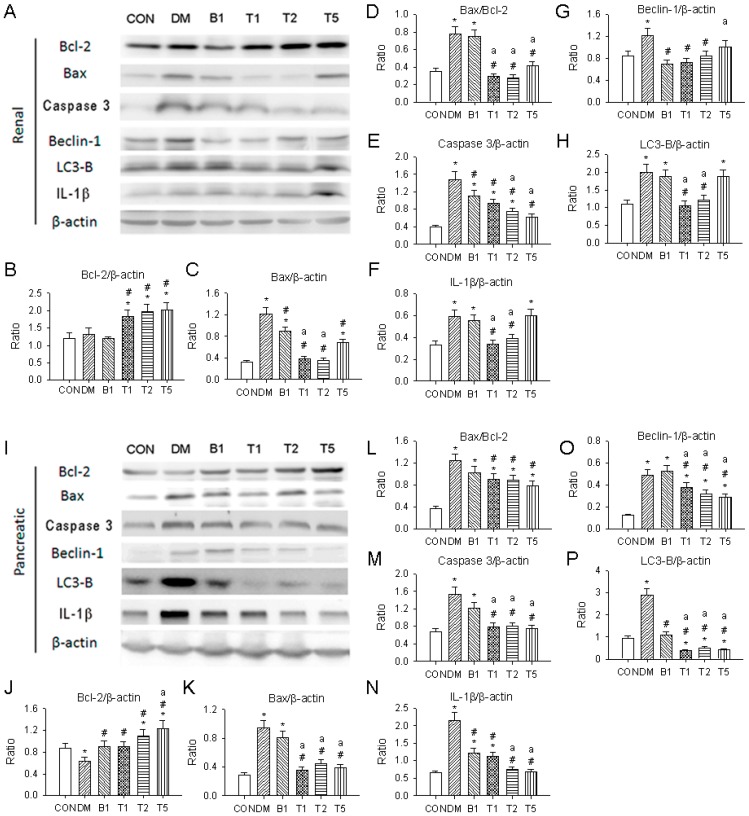
Effect of guava juice and trehalose on apoptosis-, autophagy- and pyroptosis-related proteins expression in the T2DM kidney and pancreas by western blot. Typical western blotting graphs of kidney (**A**) and pancreas (**I**) are shown; The statistical data of each protein expression and ratio are shown in the kdieny (**B**–**H**) and pancreas (**J**–**P**). Data are expressed as mean ± SEM. *n* = 3–5 each group.* *p* < 0.05 *vs.* CON. # *p* < 0.05 *vs.* DM. ^a^ *p* < 0.05 *vs.* B1.

Compared to the control kidney and pancreas, the expressions of autophagy mediated proteins, Beclin-1 and LC3-B, pyroptosis mediated proteins, IL-1β, and apoptosis related proteins, Bax, caspase 3 and Bax/Bcl-2 ratio, were significantly higher in DM and were lowered in T1, T2 and T5 (*p* < 0.05) (Figure 8).

T1, T2, T5 or B1 significantly decreased renal and pancreatic Beclin-1, LC3-B, IL-1β, caspase 3 expressions and Bax/Bcl-2 ratio. B1 rats had higher renal and pancreatic Beclin-1, LC3-B, IL-1β, and caspase 3 expression and Bax/Bcl-2 ratio when compared to T1, T2 and T5.

## 3. Discussion

We compared T1 to B1 to find out whether trehalose really had additional effect on T2DM. According to the HOMA-β result, T1 rats showed a better effect on pancreatic β cell function than B1 did. T1 rats had slightly higher HOMA-β at week 4 than week 0, while B1 rats still had low HOMA-β values. However, this protective function didn’t maintain in HOMA-IR and insulin levels. HOMA-IR could be a useful method not only diagnosing insulin resistance, but also for follow-up during the treatment of patients with T2DM [19]. There was no significant difference of HOMA-IR between T1 and B1. The addition of trehalose didn’t increase in insulin sensitivity between T1 and B1. Our data found that elevated doses of guava juice seem to increase insulin secretion and reduce HOMA-IR. These results indicated trehalose supplementation helps conserve β cell function, but not improve insulin resistance. However, guava juice might increase the sensitivity of insulin with a dose of more than 8 mL/kg BW/day (T2 and T5 groups).

Diabetic nephropathy is associated with structural abnormalities including glomerulosclerosis [7]. Our results from Masson’s staining experiments revealed marked collagen accumulation in T2DM kidneys. The renal sections showed that the major sclerosis appeared in the tubule area, rather than in the glomerular area. DM-induced sclerotic injury was efficiently rescued by guava juice treatment in T2 and T5 rats. In addition, treatment of guava juice protected hemolysis and inflammation in the kidney and cell arrangement in pancreas. 

This study proved that guava juice combined with trehalose had an efficient protective function against T2DM-induced renal and pancreatic pathology and dysfunction. Previous studies frequently stated that excess ROS amount impaired renal function and structures and increased antioxidant defense mechanism protected renal function and structures [20,21,22]. Our data displayed that the high content of quercetin in the guava juice scavenged H_2_O_2_ and HOCl, whereas trehalose selectively reduced H_2_O_2_, but not HOCl. T2DM affected the levels in OGTT, plasma insulin, HbA1c, HOMA-IR and HOMA-β, whereas these T2DM-altered parameters, except HbA1c, were significantly improved by guava and trehalose treatment. The levels of T2DM-enhanced renal ROS, 3-nitrotyrosine, caspase-3 mediated apoptosis, LC3-B mediated autophagy and IL-1β mediated pyroptosis were significantly decreased by guava juice and trehalose. Combination of trehalose and guava juice protects the pancreas and kidney in rats against T2DM-induced oxidative stress, inflammation and programmed cell death. 

Genetic disposition, aging, obesity and dietetic life style contributed to T2DM [23]. High calory consumption, high fat diet and high fructose diet were the main dietetic lifestyle behavior causing metabolic syndromes and chronic diseases. The animal model we developed in this study was relatively similar to the lifestyle of human nowadays. High fructose is associated with insulin resistance, hyperglycemia and hypertension [24]. High fructose also induced lipogenesis from liver and produces great amount of triglyceride which furthermore reduced insulin sensitivity [25]. Traditional T2DM models, such as yellow A^(vy/−)^ mice, db/db mice, were trans gene and spontaneous induced animals. They have the advantages of stability and time savings, but cost a lot. In addition, T2DM development in these kinds of animals was predominantly genetic, unlike in the human population, and also unlike the pattern of the human clinical situation [23]. A traditional and easy way to induce T1DM was by injection of streptozotocin (STZ, a glucosamine-nitrosurea compound) that damages pancreas β cells and impairs insulin secretion [9]. On the other hand, nicotinamide (NA) supplied NAD, which would be affected because of STZ-induced DNA damage [2]. Animals administered STZ and NA appeared in a closer phase to T2DM [26]. That is the reason why we used this model in our study.

Guava and its leaf have been frequently used for their anti-oxidative ability [27]. In our study, the anti-oxidative ability of guava juice was efficient in scavenging H_2_O_2_ and HOCl and this was consistent with the previous findings [27]. Furthermore, this antioxidant effect can be preserved after oral consumption. The result of *in vitro* serum ROS levels evidenced that the anti-oxidative property of guava could reduce the amount of H_2_O_2_ and HOCl. The result of guava juice on reduction of 4-HNE IHC stain in renal and pancreatic sections also confirmed the antioxidant effect. Our study also found that the addition of trehalose seemed to reinforce the anti-oxidative ability of guava. Growing *Candida albicans* cells from trehalose-deficient mutant were extremely sensitive to oxidative stress exposure (H_2_O_2_), while in wild-type cells, the same H_2_O_2_ exposure induced intracellular accumulation of trehalose and displayed a higher resistance to oxidative stress [14]. These researchers implied that trehalose could be a promising free radical scavenger for mammals’ organs [14]. In our study, serum ROS levels and 4-HNE stain in T2 and T5 rats were significantly lower than those in B1 rats. Although there was no significantly difference between B1 and T1, T1 still displayed a low ROS tendency and 4-HNE stain compared to B1. We suggest that guava juice through the high content of quercetin and trehalose itself can reduce ROS-induced oxidative injuries. Our results were consistent with the previous findings from Chen and Haddad [14].

The most important characteristic of diabetes is the appearance of hyperglycemia. In our study, there is no obvious improvement of blood glucose, because the blood glucose difference among DM rats and other rats treated with guava juice and trehalose was insignificant. In a previous report, guava showed anti-hyperglycemic effects [28]. However, the animal model was different from our model. Nevertheless, our animal model more likely represented the late phase of T2DM, because it lacked insulin compensation of β cells, which happens in the primary phase. We defined T2DM successfully for induction of FBG over 230 mg/dL with a severe hyperglycemic condition. In contrast to a previous study, T2DM was defined by FBG over 11.1 mmol/L, which is equal to 200 mg/dL, in rats injected STZ and NA [29]. In a mouse study with STZ and NA injection, plasma insulin levels stay the same as in control mice [2]. Due to these differences, the damage of T2DM in our rats could be irreparable.

We explored the role of three types of programed cell death, including apoptosis, autophagy and pyroptosis in T2DM kidneys and pancreas. Butler *et al.* [30] found β-cell deficit by the increased β-cell apoptosis in humans with T2DM and Masini *et al.* [31] indicated increased autophagy in human T2DM pancreatic β cells. The data of caspase 3 expression of renal and pancreatic sections showed that treatment of guava juice and trehalose prevented cells from apoptotic injury indicating their anti-apoptotic potential in the kidney and pancreas in our study. We also noticed the upregulation of autophagy-related protein Beclin-1/LC3-B expression in the kidneys and pancreas of DM rats in our study. Beclin-1 and LC3-B were autophagy-mediated proteins which had a central role in the formation of the autophagosome of autophagy and increased during periods of cell stress [32]. In addition, our data further indicated that DM-enhanced IL-1β, a pyroptosis biomarker, was depressed by guava juice and trehalose in the pancreas and kidney. Guava juice itself could help protect cell from apoptosis, pyroptosis and autophagy in the T2DM kidney and pancreas. The supplement of trehalose helped to enhance the protective function from apoptosis, autophagy and pyroptosis in the kidney and pancreas.

Our data in this study showed that T5, which groups of rats had consumed the highest dose of guava juice and trehalose per day, didn’t provide the best protective function. According to our results, the values of water intake, Bax expression in kidney and HOMA-β in T5 were not even better than in the T1 and T2 groups. We suggest that the protective effect of guava juice and trehalose would be strengthened dose-dependently. However, fruit juice may have some differences from fruit itself. This may be due to a lower percentage of fibers and a higher sugar load in the guava juice than with fruit itself. High consumption of fruit juices may contribute to a higher dietary glycemic load which has been positively associated with DM [33]. Therefore, in our study, we wondered whether the dose of guava juice was up to 20 mL/kg BW/day with trehalose 1 g/kg BW/day may be overloading. The dose consumed by T2 rats may have the best protective function toward T2DM.

In conclusion, the anti-oxidative, anti-inflammatory and antidiabetic properties of guava, possibly through quercetin action, were used to inhibit T2DM-induced inflammation and oxidative injury. The addition of trehalose helped to further prevent cell death from apoptosis, autophagy and pyroptosis, to conserve β cell function and to enhance the protective ability. 

## 4. Experimental Section

### 4.1. Guava Extraction

The guava juice used in this study was made from two kinds of guava harvested in Taiwan, Thailand guava (G1) and pearl guava (G2). A fifty gram edible portion (the seed portion was removed) of fresh fruits was blended with 150 mL H_2_O or 50% ethanol. After storing the extract at room temperature (25 °C) for 24 h, we filtered it through gauze, and then Whatman No. 1 filter paper [10]. A Strata C18-E column (6 mL) was first rinsed by 12 mL methanol, and then rinsed by 12 mL ddH_2_O. We added the 24 mL water extract or 48 mL ethanol extract (diluted two times with ddH_2_O) to the column. After all the extract was eluted, we washed the column with 12 mL ddH_2_O. We rinsed out the final concentrated extract with 12 mL methanol. Guava water extract of G1 (GWE1), G2 (GWE2) and guava ethanol extract of G1 (GEE1), G2 (GEE2) were stored at −20 °C.

### 4.2. LC/MS

We used an Ultimate 3000 HPLC system comprising a HPLC pump, diode array absorbance detector to analyze tested samples (guava juice extractions) at the range from 200 to 600 nm (Thermo Finnigan, San Jose, CA, USA). Separation was carried out using a 150_2.0 mm i.d. 4 mm Synergi RP-Max column (Phenomenex, Macclesfield, UK) eluted with a gradient over 40 min of 10%–80% acetonitrile in 0.1% aqueous formic acid at a flow rate of 0.2 mL/min. After passing through the flow of the diode array detector, the column eluate was directed to a LXQ ion trap mass spectrometer fitted with an electrospray interface (Thermo Finnigan). The analyses utilized the negative ion mode as this provided the best limits of detection for quercetin. Capillary temperature was set at 325 °C, sheath gas and auxiliary gas were 30 and 15 arb, respectively, and source voltage was 3.5 kV.

### 4.3. Animals

The experiments were performed with 7-week old female Wistar rats purchased from BioLASCO Taiwan Co. Ltd. (Taipei, Taiwan) that were housed at the Experimental Animal Center, National Taiwan Normal University, at a constant temperature and with a consistent light cycle (light from 07:00 to 18:00 O’clock). All surgical and experimental procedures were approved by National Taiwan Normal University Animal Care and Use Committee and were in accordance with the guidelines of the National Science Council of Republic of China (NSC 1997). 

### 4.4. Induction of T2DM

Group CON consumed standard diet and normal water. The other five groups were induced to be T2DM by consuming a high fructose diet for 8 weeks and then intraperitoneal (i.p.) injection of nicotinamide (NA, Sigma, Missouri, MO, USA, 230 mg/kg) and streptozotocin (STZ, Sigma, 65 mg/kg). The high fructose includes 21% fructose water and 60% fructose diet (TD. 89247, Harlan Teklad, Frederick, MD, USA). The nutrition information of 60% fructose diet is showed in the following column. After consuming high fructose diet for 8 weeks, the rats were injected NA and STZ, and these rats with fasted blood glucose (FBG) higher than 230 mg/dL were recognized to be T2DM.

### 4.5. Animal Treatment

Different treatments among these groups are showed as following. Animals were randomly assigned to six groups: control (CON) group; type II DM (DM) group; T2DM with 4 mL/kg BW of guava juice with 2 mL/kg BW of trehalose (T1); T2DM with 8 mL/kg BW of guava juice with 4 mL/kg BW of trehalose (T2); T2DM with 20 mL/kg BW of guava juice with 1 mL/kg BW of trehalose (T5) and T2DM with 4 mL/kg BW of guava juice without trehalose (B1). Different groups of rats were given different doses of 40% guava juice by gavage for 4 weeks.

### 4.6. Determine Blood Glucose

Blood glucose concentrations (mg/dL) were determined by Bayer Ascencsia ELITE XL (Bayer Healthcare, Whippany, NJ, USA). FBG was measured after fasted overnight (14 h).

### 4.7. Determine Insulin Levels

Blood samples were collected into heparinized tubes from the femoral artery before and 30 and 60 min after oral gavage for plasma insulin determination. Insulin levels were determined by Mercodia Rat Insulin ELISA kit (Mercodia, Uppsala, Sweden). Insulin data indicated with area under curve (AUC, min × μg/L) of this experiment were calculated by the trapezoidal rule during time interval 0–60 min.

### 4.8. HOMA-IR and HOMA-β

Homeostasis model assessment of IR (HOMA-IR) is an indicator of insulin resistance in diabetic patients [19]. HOMA-β represents the function of β cell in pancreas and insulin secretion index. HOMA-IR is calculated by the formula: [fasted insulin (μU/mL) × fasting blood glucose (mg/dL)]/405. HOMA-β is calculated by the formulae: (360 × fasted insulin (μU/mL)/(fasting blood glucose (mg/dL) minus 63). We calculated these two values at week 0 (the beginning of the 4 week guava juice treating experiment) and week 4 (the end of the 4-week guava juice treating experiment).

### 4.9. Glucose Tolerance Test

#### 4.9.1. Intra Venous Glucose Tolerance Test (IVGTT)

After fasted overnight, the animals were anaesthetized with avertin (200 mg/kg BW, i.p.) (Sigma). The carotid and jugular veins were intubated with PE50 tube to collect the blood sample for blood glucose testing and to inject glucose (0.5 g/kg). We injected trehalose (0.5 g/kg) to compare the capacity to lift blood glucose with glucose. The values of blood glucose were recorded before the injection, and 1, 5, 10, 20, 30, 50, and 75 min after treatment.

#### 4.9.2. Oral Glucose Tolerance Test (OGTT)

OGTT were performed after fasted overnight. An oral glucose load (2 g/kg BW) was treated by gavage. Blood samples were collected to test blood glucose levels from the tail vein. These samples were collected before the gavage (0 min) and 30, 60, 90 and 120 min after it. We also determined the tolerance of guava juice to determine blood glucose change after consumption of guava juice.

### 4.10. Determine HbA1c

HbA1c, glycated hemoglobin *A1c*, was measured by Cation-Exchange HPLC (HLC-723 G7 HPLC analyzer, Tosoh Bioscience, Tessenderlo, Belgium). 

### 4.11. In Vitro ROS Level

The two major ROS generated from activated neutrophils via the myeloperoxidase system are hydrogen peroxide (H_2_O_2_) and hypochlorite (HOCl), both of which can initiate inflammation [20]. In brief, chemiluminescent signals emitted from the test mixture of phosphate-buffered saline (PBS) (50 mmol/L, pH 7.4) containing 50 μL of H_2_O_2_ (0.03 %) or HOCl (0.0005 %) were amplified by 0.5 mL of luminol (5-amino-2,3-dihydro-1,4-phthalazinedione, Sigma Chemical Co., St. Louis, MO, USA) was measured with a ultrasensitive chemiluminescent analyzer (CLA-ID3; Tohoku Electronic Industrial Co., Ltd., Sendai, Japan). We evaluated the guava juice and trehalose effect on the enhanced chemiluminescent signals from the H_2_O_2_-luminol-PBS mixture or HOCl-luminol-PBS mixture. The assay was performed in duplicate for each sample, and total CL counts in 600 s were calculated by integrating the area under the curve.

### 4.12. In Vivo ROS Level

Real-time O_2_^−^ generation was measured from an amplified chemiluminescent signal of the kidney surface *in vivo*, which was demonstrated previously from our laboratory [20]. In brief, an O_2_^−^ probe, 2-methyl-6-(4-methoxyphenyl)-3,7-dihydroimidazo-[1,2-*a*]-pyrazin-3-one-hydrochloride (MCLA; TCI-Ace, Tokyo Kasei Kogyo Co. Ltd., Tokyo, Japan) was infused intravenously throughout the experiment at 0.2 mg/mL/h using a Chemiluminescence Analyzing System (CLA-ID3, Tohoku Electronic Industrial Co., Ltd.). The rat was maintained on a circulating water pad at 37 °C during photon detection. For excluding photon emission from sources other than the kidney, the animal was housed in a dark box with a shielded plate. Only the left kidney was left unshielded and was positioned under a reflector, which continuously directed the MCLA-enhanced chemiluminescent signal from the exposed kidney surface onto the detector area. The chemiluminescence counts were recorded every 10 sec and the total O_2_^−^ value was measured using the area under the detected photons-time curve. The chemiluminescent signal obtained from 0.2 mL saline in 1 mL of MCLA (0.2 mg/mL) was regarded as the negative control. The signal obtained from 0.2 mL xanthine (0.75 mg/kg body weight)/xanthine oxidase (24.8 mU/kg body weight) in 1 mL of MCLA (0.2 mg/mL) was regarded as the positive control.

### 4.13. Immunohistochemistry (IHC)

Deparaffinized tissue sections were used to perform IHC [20]. Primary antibodies included mouse anti IL-1β (1:1000; Cell Signaling Technology, Danvers, MA, USA), rabbit anti Caspase 3 (1:500; Cell Signaling Technology), and rabbit anti 4-HNE (4-hydroxynonenal; 1:500; Alpha Diagnostic, San Antonio, TX, USA). We immersed slides in DAB (ImmPACT DAB Peroxidase Substrate; Vector, Burlingame, California, CA, USA) for 3–5 s, washed with ddH_2_O, and immersed slides in hematoxylin for 5 min. The sections were dehydrated in ethanol series and were mounted in mounting medium (Leica, Wetzlar, Germany).

### 4.14. Masson’s Stain

Paraffin sections (5 μm) in the kidney sections were stained with Masson’s trichrome or HE stain.

### 4.15. Fluorescent Stain

Deparaffinized tissue sections were submitted to antigen retrieval step. After 15 min of antigen retrieval step, sections were blocked for non-specific binding with 5% bovine serum albumin (Sigma-Aldrich, St. Louis, MO, USA) for 1 h at room temperature and incubated with the primary antibodies, including bs-2912R-Cy5 (rabbit anti MAP1A/MAP1B LC3 B polyclonal antibody, Cy5 conjugated) (1:500, Bioss, Woburn, MS, USA) and bs-0081R-FITC (rabbit anti-Caspase-3 polyclonal antibody, FITC conjugated) (1:500) for 18 h at 4 °C. The nuclear stain was dyed with Hoechst33342 (1:1000; Sigma-Aldrich) for 1 h at room temperature. After washing with PBS, tissue sections were mounted with mounting medium (Leica). The slides were scanned by a Leica TCS SP3 laser confocal microscope.

### 4.16. Western Blotting 

Protein expression in kidney and pancreas were detected by western blotting [21,22]. Primary antibodies included IL-1β, LC3-β (MBL, Naka-ku, Nagoya, Japan), caspase 3, Bax, Bcl-2, Beclin-1 (Cell Signaling Technology), and β-actin (Sigma-Aldrich). Secondary antibodies included HRP-conjugated rabbit anti-mouse IgG, HRP-conjugated donkey anti-goat IgG, and HRP-conjugated goat anti-rabbit IgG (all for 1:10,000; all from Sigma Aldrich).

### 4.17. Statistical Analysis

All values are expressed as mean ± standard error mean. For comparisons of group data, two-way analysis of variance and then the Student’s unpaired *t*-test were conducted. *p* < 0.05 was considered to indicate statistical significance.

## Abbreviations

The following abbreviations are used in this manuscript:

4-HNE: 4-Hydroxynonenal
AUC: Area under curve
AGE: Advanced glycation end products
B1: T2DM with 4 mL/kg BW of guava juice without trehalose
CON: Control group
FBG: Fasted blood glucose
G1: Thailand guava
G2: Pearl guava
GWE1: Guava water extraction of G1
GWE2: Guava water extraction of G2
GEE1: Guava ethanol extraction of G1
GEE2: Guava ethanol extraction of G2
HbA1c: Glycated hemoglobin *A1c*
HOMA-IR: Homeostasis model assessment of insulin resistance
HOMA-β: Homeostasis model assessment of the function of β cell in pancreas and insulin secretion index
IHC: Immunohistochemisty
IVGTT: Intra venous glucose tolerance test
IR: Insulin resistance
MCLA: 2-Methyl-6-(4-methoxyphenyl)-3, 7-dihydroimidazo-(1,2-*a*)-pyrazin-3-one hydrochloride
NA: Nicotinamide
OGTT: Oral glucose tolerance test
ROS: Reactive oxygen species
STZ: Streptozotocin
T1: T2DM with 4 mL/kg BW of guava juice with 2 mL/kg BW of trehalose
T2: T2DM with 8 mL/kg BW of guava juice with 4 mL/kg BW of trehalose
T5: T2DM with 20 mL/kg BW of guava juice with 1 mL/kg BW of trehalose
T2DM: Type II diabetes
